# Altered Functional Brain Connectivity in Mild Cognitive Impairment during a Cognitively Complex Car Following Task

**DOI:** 10.3390/geriatrics3020020

**Published:** 2018-04-19

**Authors:** Megan A. Hird, Nathan W. Churchill, Corinne E. Fischer, Gary Naglie, Simon J. Graham, Tom A. Schweizer

**Affiliations:** 1 Neuroscience Research Program, Keenan Research Centre for Biomedical Science, St. Michael’s Hospital, Toronto, ON M5B 1T8, Canada; m.hird@mail.utoronto.ca (M.A.H.); nchurchill.research@gmail.com (N.W.C.); FischerC@smh.ca (C.E.F.); 2 Department of Medicine, University of Toronto, Toronto, ON M5S 1A8, Canada; 3 Department of Psychiatry, Division of Geriatric Psychiatry, St. Michael’s Hospital, University of Toronto, Toronto, ON M5B 1W8, Canada; 4 Department of Medicine and Rotman Research Institute, Baycrest Health Science, Toronto, ON M6A 2E1, Canada; gnaglie@baycrest.org; 5 Department of Medicine and Institute of Health Policy, Management and Evaluation, University of Toronto, Toronto, ON M5S 1A8, Canada; 6 Department of Research, Toronto Rehabilitation Institute, University Health Network, Toronto, ON M5G 2A2, Canada; 7 Physical Sciences Platform, Sunnybrook Research Institute, Toronto, ON M4N 3M5, Canada; sgraham@sri.utoronto.ca; 8 Department of Medical Biophysics, University of Toronto, Toronto, ON M5G 1L7, Canada; 9 Department of Surgery, Neurosurgery Division, University of Toronto, Toronto, ON M52 3H7, Canada; 10 Institute of Biomaterials and Biomedical Engineering, University of Toronto, Toronto, ON M5S 3G9, Canada

**Keywords:** mild cognitive impairment, driving, fMRI, functional connectivity

## Abstract

Mild cognitive impairment (MCI) can affect multiple cognitive abilities, leading to difficulty in performing complex, cognitively demanding daily tasks, such as driving. This study combined driving simulation and functional magnetic resonance imaging (fMRI) to investigate brain function in individuals with MCI while they performed a car-following task. The behavioral driving performance of 24 patients with MCI and 20 healthy age-matched controls was compared during a simulated car-following task. Functional brain connectivity during driving was analyzed for a separate cohort of 15 patients with MCI and 15 controls. Individuals with MCI had minor difficulty with lane maintenance, exhibiting significantly increased variability in steering compared to controls. Patients with MCI also exhibited reduced connectivity between fronto-parietal regions, as well as between regions involved in cognitive control (medial frontal cortex) and regions important for visual processing (cuneus, angular gyrus, superior occipital cortex, inferior and superior parietal cortex). Greater difficulty in lane maintenance (i.e., increased steering variability and lane deviations) among individuals with MCI was further associated with increased connectivity between the posterior cingulate cortex (PCC) and inferior frontal gyrus, as well as increased intra-cerebellar connectivity. Thus, compared to cognitively healthy controls, patients with MCI showed reduced connectivity between regions involved in visual attention, visual processing, cognitive control, and performance monitoring. Greater difficulty with lane maintenance among patients with MCI may reflect failure to inhibit components of the default-mode network (PCC), leading to interference with task-relevant networks as well as alterations in cerebellum connectivity.

## 1. Introduction

Mild cognitive impairment (MCI) is often conceptualized as being a continuum, between typical age-related cognitive changes and the more moderate to severe deficits characteristic of Alzheimer’s disease (AD) and related dementia. The cognitive presentation and progression of patients with MCI is highly heterogeneous. Although some individuals with MCI ultimately progress to AD or related dementia, many maintain their clinical status or improve and revert to normal healthy aging. The original diagnostic criteria outlined by Petersen et al. [1] emphasized that individuals with MCI demonstrate “normal” performance on activities of daily living (ADLs) and instrumental activities of daily living (IADLs). However, more research has suggested that individuals with MCI may exhibit modest difficulties or impairments when performing more complex daily activities, such as managing finances and driving [2,3,4,5]. 

Driving is one of the most cognitively complex daily activities, requiring the integration of multiple cognitive functions (including attention, executive function, visual spatial ability, and memory) and the engagement of a spatially extensive brain network (including frontal, parietal, motor, and cerebellar regions) [6,7,8,9,10]. Previous on-road [11] and simulator-based studies [12,13,14,15] have reported that patients with MCI exhibit minor difficulties when driving, rather than definitive impairment, particularly with lane maintenance [11,15] and car following [13].

Given the mild nature of cognitive and functional difficulties associated with MCI, it is particularly important to understand brain changes associated with MCI during complex daily activities, including driving. Identifying early functional and structural markers in the disease process continues to provide a greater understanding of the etiology and disease progression of AD [16]. Given that changes in brain function may precede structural changes [16], functional magnetic resonance imaging (fMRI), which measures brain activity based on fluctuations in blood oxygen levels, may provide important insights into the pathophysiology of MCI. For example, functional connectivity analyses measure temporal synchronization of fMRI signals between different brain regions, as a measure of information sharing [17,18,19], with greater functional connectivity potentially indicating greater functional integration between regions. 

Multiple studies have supported the utility of task-based functional connectivity in multiple neurological populations, including MCI [19,20,21,22]. Bajo et al. [19] reported increased interhemispheric connections as well as reduced anterior-posterior functional connectivity during a memory task using magnetoencephalography. Bokde et al. [20] observed both reduced and increased functional connectivity between the middle frontal gyrus and multiple brain regions during a face-matching task using fMRI. However, the tasks utilized in these studies were relatively simplistic and assessed single cognitive domains. Given the subtle nature of MCI, it is important to progress from this work towards research that involves more complex and multi-faceted daily tasks, such as driving, to provide more sensitive characterization of the cognitive deficits of individuals with MCI in relation to healthy individuals. The present study addresses this need by using fMRI and driving simulation to identify patterns of functional connectivity associated with MCI and MCI-related driving difficulty. The specific aims of this study were to identify: (1) aspects of driving difficulty among patients with MCI during a cognitively complex simulated car following task; (2) alterations in functional connectivity in MCI relative to cognitively healthy adults during the simulated car following task; and (3) alterations in functional connectivity associated with increased difficulty with lane maintenance among individuals with MCI.

## 2. Materials and Methods 

### 2.1. Behavioral Performance of Individuals with MCI on Car Following Task 

#### 2.1.1. Participants

The study was approved by the Research Ethics Board at St. Michael’s Hospital, Toronto, and all participants provided written informed consent prior to participating. Twenty-nine (29) individuals with MCI were recruited from the Memory Disorders Clinic at St. Michael’s Hospital. All participants with MCI met the National Institute on Aging-Alzheimer’s Association criteria [23] for MCI (both single and multiple domain MCI). Specifically, all patients had (1) concern regarding change in cognition; (2) objective impairment in one or more cognitive domains; (3) preserved independence in functional ability; and (4) no presence of dementia [23]. All patients were diagnosed by a geriatric psychiatrist based on a comprehensive history, clinical neuroimaging (CT, MRI, and/or SPECT), and cognitive testing, including the Behavioral Neurological Assessment (BNA), the Montreal Cognitive Assessment (MoCA), and the Mini-Mental Status Examination (MMSE). 

Cognitively healthy control participants matched on age and driving experience (*n* = 24) were recruited from the community, through St. Michael’s Hospital, the University of Toronto Senior Alumni Association, and Baycrest Health Sciences. All controls reported no memory concerns and scored ≥26 on the MoCA. Exclusion criteria, for both MCI and control groups, included a history of substance abuse, presence of a neurological condition (e.g., Parkinson’s disease, multiple sclerosis, etc.), presence of a psychiatric condition (e.g., bipolar disorder, schizophrenia, etc.), chronic use of medications that could alter cognition, or severe motor abnormality. At the time of testing, all participants held a full (Ontario G) driver’s license and met the vision standards outlined by the Ontario Ministry of Transportation. 

#### 2.1.2. Car Following Task

The car following task was administered using a portable driving simulator (STISIM Drive^®^, Logitech G25 model), which included a steering wheel, accelerator pedal, brake pedal, and signaling system. The task required participants to follow a car while maintaining a consistent distance from the lead vehicle. The speed of the lead vehicle followed a continually-varying sinusoidal pattern, ranging from 50 to 90 km/h (one period of oscillation = 22.5 s). Car following tasks require a high degree of sustained attention and error monitoring [10]. 

Five (5) patients with MCI and 4 healthy control participants experienced simulator sickness (17% of total sample) and were unable to complete the car following task. This frequency of simulator sickness is consistent with previous literature [24,25]. Thus, 24 patients with MCI and 20 healthy control participants completed the study.

#### 2.1.3. Statistical Analysis of Task Performance

Multiple variables of interest were recorded during the car following task: centerline crossings, road edge excursions, speed exceedances, lane deviations (centerline crossings + road edge excursions) total errors (lane deviations + speed exceedances), speed variability (standard deviation in speed), lane variability (standard deviation in lane position, SDLP), and steering variability (standard deviation (SD) in steering). These variables were compared between individuals with MCI and healthy controls using the Mann-Whitney U test.

### 2.2. Functional Brain Connectivity during Car Following Task

#### 2.2.1. Participants

For the fMRI portion of the study, 15 individuals with MCI were recruited from the Memory Disorders Clinic at St. Michael’s Hospital. All patients were formally diagnosed by a geriatric psychiatrist as described above. Fifteen (15) healthy control participants were recruited from the community. Although there was some overlap in the participants who participated in the behavioral and fMRI portions of the study, the majority of the fMRI sample represented a separate cohort of participants. Patients and controls were group-matched on age, sex, and years of driving experience. The inclusion/exclusion criteria for this portion of the study were the same as for the previous group; however, potential participants with contraindications to MRI (e.g., claustrophobia, metal implants) were also excluded during recruitment.

#### 2.2.2. fMRI of the Car Following Task

Participants completed the same car following task described in Section 2.1.2 while lying supine within the magnet bore of a 3 T MRI system (Magnetom Skyra, Siemens, Erlangen, Germany). During the task, they interacted with a custom fMRI-compatible driving simulator that included a steering wheel at their waist, accelerator and brake pedals at their feet, and a mirror attached to the head coil of the MRI system. Participants viewed the simulation environment through the mirror angled at a screen that was illuminated by an MRI-compatible projector (Avotec, Stuart, FL, USA). The set-up is described in greater detail by Kan et al. [26]. 

Structural images were acquired by T1-weighted Magnetization Prepared Rapid Acquisition Gradient Echo (MPRAGE: inversion time (TI)/echo time (TE)/repetition time (TR) = 1090/3.55/2300 ms, flip angle (FA) = 8°, 192 sagittal slices with field of view (FOV) = 240 × 240 mm, 256 × 256 matrix, 0.9 mm slice thickness, 0.9 mm × 0.9 mm in-plane resolution, bandwidth (BW) = 200 Hertz/pixel (Hz/px)). The fMRI data were acquired by multi-slice T2*-weighted echo planar imaging (EPI: TE/TR = 30/2000 ms, FA = 70°, 32 oblique-axial slices acquired interleaved ascending, with FOV = 200 × 200 mm, 64 × 64 matrix, 4.0 mm slice thickness with 0.5 mm gap, 3.125 × 3.125 in-plane resolution, BW = 2298 Hz/px). 

#### 2.2.3. fMRI Preprocessing

Data preprocessing and analyses of brain activation were performed using a hybrid pipeline, developed by Churchill et al. [27] which uses tools from Analysis of Functional Neuroimages (AFNI) package [28], the Functional Magnetic Resonance Imaging of the Brain (FMRIB) Software Library (FSL) package [29], and algorithms developed in the laboratory. The pipeline involved rigid-body motion correction (AFNI 3dvolreg), slice-timing correction (AFNI 3dTshift), removal of outlier scan volumes (nitrc.org/projects/spikecor), spatial smoothing with a 6 mm Full Width at Half Maximum (FWHM) isotropic 3D Gaussian kernel (AFNI 3dmerge), along with regression of linear-quadratic trends and motion parameters as nuisance covariates. Data-driven correction for physiological noise was performed using the data-driven PHYCAA+ algorithm (nitrc.org/projects/phycaa_plus) to de-emphasize areas with non-neural signal, followed by regression of white matter signal, done after spatial normalization (see below). The FSL flirt algorithm was used to perform the rigid-body transform of the mean fMRI volume for each participant to their T1-weighted anatomical image and the 12-parameter affine transformation of the anatomical image for each participant to the MNI1152 (Montreal Neurological Institute) template [30]. The transformation matrices were concatenated, and the net transform was applied to the fMRI data, which were kept at the original voxel resolution. To remove white matter signal, the FMRIB FSL fast algorithm was used to segment the T1-weighted MPRAGE anatomical images for each subject, and white matter maps were transformed into MNI152 template space, after which a mean group level probabilistic white matter atlas was generated. For each participant, the mean BOLD time series was computed within white matter regions (*p* > 0.95) and regressed from all voxels.

#### 2.2.4. Functional Connectivity Analysis

The automatic anatomical labelling (AAL) atlas [31] was used to parcellate the brain into a set of *n* = 116 distinct regions of interest (ROIs). For each subject, after z-scoring voxel time series (i.e., subtracting the temporal mean and dividing by standard deviation) the mean time series z signal was determined for each ROI. The full 116 × 116 functional connectivity matrix was then obtained by computing the Pearson correlations for each pair of ROI time series z signals. Tests for any group-level effects of MCI status and driving performance were computed independently for each of the 6670 unique pairwise connections as follows:

##### Differences between MCI and Healthy Older Controls

The mean difference in connectivity for MCI, relative to healthy controls was computed for each of the pairwise connections. Effect sizes were determined by performing non-parametric bootstrap analyses: random resampling (with replacement) was performed and the mean difference measured for each resample (1000 iterations). An empirical *p*-value was obtained based on the fraction of resamples that did not overlap zero, adjusted for multiple comparisons at a false discovery rate of 0.05. For significant connections, the effect size was reported as a bootstrap ratio (bootstrap mean/standard error). 

##### Relationship with Lane Maintenance Behavior in MCI

Within the MCI group only, the correlation was computed between connectivity values and two measures of lane maintenance: number of lane deviations and standard deviation in steering. The computations were performed for each pairwise connection, with *p*-value and effect size obtained via non-parametric bootstrap resampling, as outlined above.

## 3. Results

### 3.1. Behavioral Performance of Individuals with MCI on Car Following Task

Demographic information for the 44 participants is reported in Table 1. There were no significant differences between patients with MCI and healthy controls on any demographic variables (including age, education, sex, driving experience, self-reported accidents). Individuals with MCI scored significantly lower on the MoCA compared to healthy controls (mean scores of 27.9 vs. 23.8, *p* < 0.001). 

The results of the car following analysis (Table 2) revealed that patients with MCI and healthy control participants followed the lead vehicle to a similar degree (i.e., having comparable standard deviation in lane position, speed, and range from the car). However, individuals with MCI exhibited significantly greater steering variability compared to healthy controls. No differences emerged between the two groups for total errors or individual errors committed (centerline crossings, road edge excursions, collisions, speed exceedances). 

### 3.2. Functional Brain Connectivity during Car Following Task

The demographic characteristics of the individuals with MCI who participated in the fMRI portion of the study were similar to those who completed the behavioral session, including age, education, driving experience, and cognitive performance (Table 3). There was a greater proportion of male MCI patients who completed the fMRI session compared to the behavioral session (80% vs. 58.3%); Importantly, the percentage of males was similar for the MCI (*n* = 12) and healthy control (*n* = 11) groups in the fMRI session (80% vs. 73%). 

#### 3.2.1. Differences between MCI and Cognitively Healthy Drivers

**Figure 1:**
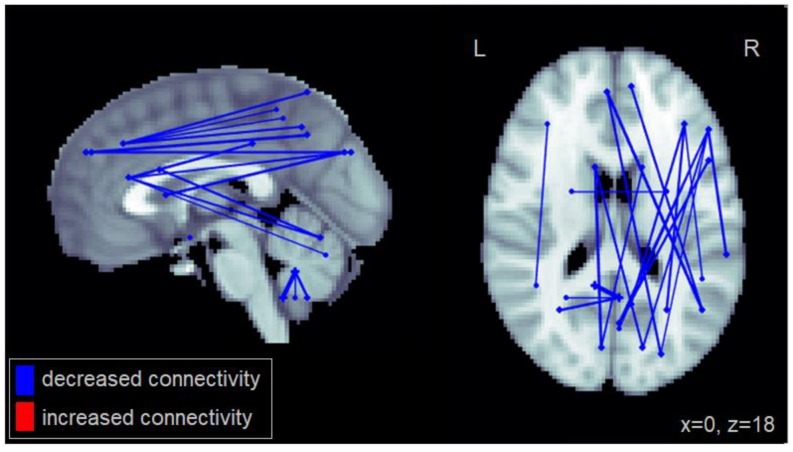
Altered functional connectivity observed for the MCI group compared to the healthy control group. Blue lines denote functional connections with reduced connectivity in MCI patients, red lines denote increased connectivity (significant at FDR = 0.05). Connections are shown as projections in the sagittal and axial plane, with MNI anatomical underlays (slices at x = 0 and z = 18 respectively).

Compared to healthy controls, patients with MCI generally showed reduced functional connectivity, particularly long-distance connections between frontal and posterior cortical regions (Figure 1). Specifically, the MCI group exhibited reduced connectivity between the middle frontal cortex and the superior and inferior parietal cortex; between the superior medial frontal cortex and the superior occipital cortex and the angular gyrus; and between the inferior frontal gyrus and the cuneus as well as the superior occipital cortex. Reduced connectivity was also observed between the inferior orbitofrontal cortex and the cerebellum, within the cerebellum, and between the caudate and the cuneus. The complete set of regions showing reduced connectivity among patients with MCI is reported in Table A1. 

#### 3.2.2. Functional Connectivity and Lane Maintenance Behavior in the MCI Group

**Figure 2:**
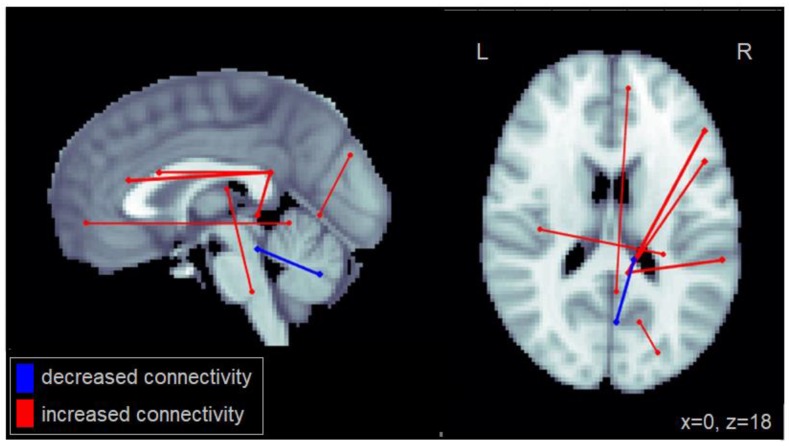
Altered functional connectivity associated with number of lane deviations observed among patients with MCI. Blue lines denote functional connections with reduced connectivity associated with greater lane deviations, red lines denote increased connectivity (significant at FDR = 0.05). Connections are shown as projections in the sagittal and axial plane, with MNI anatomical underlays (slices at x = 0 and z = 18 respectively).

An increased number of lane deviations (centerline crossings + road edge excursions) among patients with MCI was associated primarily with increased connectivity between the right posterior cingulate cortex (PCC), a region of the default mode network (DMN), and regions within the right inferior frontal gyrus as well as the right middle frontal cortex (Figure 2). Furthermore, increased lane deviations were associated with reduced connectivity between cerebellar regions. The complete set of regions showing alterations in connectivity among patients with MCI related to increased lane deviations is reported in Table A2.

**Figure 3:**
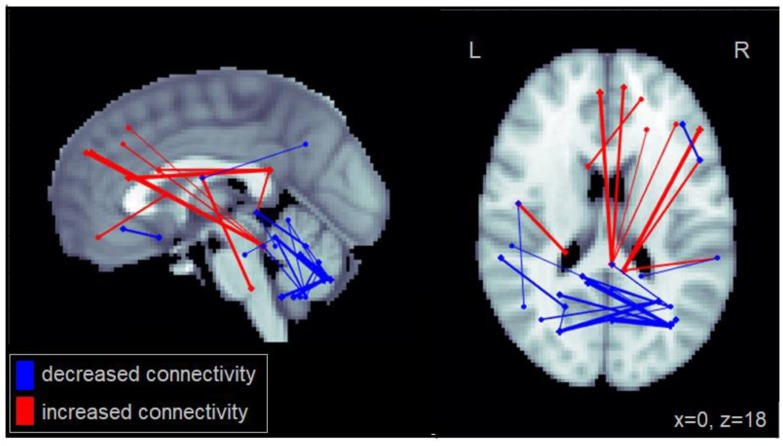
Altered functional connectivity associated with standard deviation in steering observed among patients with MCI. Blue lines denote functional connections with reduced connectivity associated with greater lane deviations, red lines denote increased connectivity (significant at FDR = 0.05). Connections are shown as projections in the sagittal and axial plane, with MNI anatomical underlays (slices at x = 0 and z = 18 respectively).

Similarly, increased steering variability (SD in steering) among patients with MCI was associated with increased connectivity between the right PCC and the right frontal gyrus as well as the right middle temporal cortex (Figure 3). Increased steering variability was also associated with reduced connectivity between cerebellar regions. This number of significantly reduced intra-cerebellar connections for steering variability was greater than that observed for lane deviations. Furthermore, increased steering variability was associated with reduced connectivity between the cerebellum and the middle and inferior temporal cortex. The complete set of regions showing alterations in connectivity among patients with MCI related to increased lane deviations is reported in Table A3. 

## 4. Discussion

Despite the subtle nature of MCI, individuals with this neurological condition may experience modest functional difficulties or impairments when performing more complex daily activities, including driving [2,3,4,5,11]. To our knowledge, the present work is the first to use fMRI to characterize the relationship between MCI, brain functional connectivity, and simulated driving performance. Consistent with previous research, patients with MCI had minor difficulty with lane maintenance, exhibiting increased steering variability compared to healthy controls. Neuroimaging results suggest that patients with MCI exhibited reduced functional connectivity between regions within the frontal cortex and posterior cortical areas during a cognitively complex car following task, compared to cognitively healthy drivers. Furthermore, among individuals with MCI, increased difficulty with lane maintenance (including lane deviations and steering variability) was associated with increased connectivity between the PCC and frontal brain regions, as well as reduced connectivity within the cerebellum.

The relative subtlety of differences in driving performance associated with MCI is an important finding that agrees with existing driving literature [11,12,13,14,15]. Furthermore, previous work supports that individuals with MCI may be at risk of driving difficulty during car following tasks [13] and across measures of lane maintenance [11,15]. 

Differences in functional connectivity were also observed between individuals with MCI and cognitively healthy drivers. Patients with MCI showed reduced functional connectivity between multiple brain areas, particularly between regions within the frontal cortex and posterior regions involved in visuospatial processing. Specifically, individuals with MCI showed reduced fronto-parietal connectivity. These connections play an important role in visuospatial attention [32] and are recruited during cognitively demanding aspects of driving [9]. Furthermore, individuals with MCI showed reduced connectivity between regions involved in visual processing (R cuneus, R angular gyrus, R superior occipital cortex, L and R inferior and superior parietal cortex) and the medial frontal cortex as well as the right inferior frontal gyrus. The latter two areas are involved in cognitive control and performance monitoring [33]. Reduced connectivity was also observed between the caudate, which is important for response switching and goal-directed action [34] and the cuneus. These reductions in functional connectivity suggest that patients with MCI have reduced information sharing between regions involved in visual attention, visual processing, cognitive control, and performance monitoring. Previous fMRI and car following studies have reported the engagement of parietal, occipital, and prefrontal brain regions during successful task performance [10,35]. Combined with present results, this suggests that task-critical brain regions are the most affected among patients with MCI. Reduced connectivity between these regions may reflect greater task difficulty, potentially leading to minor decrement in task performance. 

To investigate inter-individual variability in performance within the MCI group [11,15], functional connectivity was also investigated as a function of two measures of lane maintenance—lane deviations and steering variability (SD in steering wheel input). Individuals with MCI who committed more lane deviations showed increased connectivity between the PCC, and the inferior frontal gyrus, as well as the middle temporal cortex. Furthermore, these patients showed increased intra-cerebellar connectivity. A similar pattern was observed for steering variability, with additional reduced connectivity between the cerebellum and the middle and inferior temporal cortex. 

The PCC is an important component of the DMN, a functional brain network that exhibits a pattern of increased activation and greater functional connectivity when the brain is at rest. This network, which includes the PCC, medial prefrontal cortex, and the medial, lateral, and inferior parietal cortex, tends to show deactivation during task performance [36]. Previous research suggests that attenuation of the PCC may be important for focusing attention during task execution [36]. However, current results showed that increased connectivity between the PCC and the inferior frontal gyrus, the middle frontal cortex, and middle temporal cortex was associated with difficulty with lane maintenance among patients with MCI. This increased connectivity with the PCC, a region of the DMN, during task performance may indicate a deficit in top-down attentional control [36,37,38]. Specifically, when executing a task, the low frequency activity of the DMN can persist under certain circumstances (e.g., brain pathology) and compete with task-specific neural processes [38]. Previous research has suggested that reduced activation in the PCC is associated with better performance on a sustained attention task [39]. This is consistent with current results, which suggest that greater difficulty in task performance was associated with increased connectivity between the PCC and frontal as well as temporal regions. Therefore, some individuals with MCI may exhibit failed or decreased attenuation of components of the DMN (e.g., the PCC), interfering with task-relevant networks and leading to attentional lapses, and consequently increased errors in lane maintenance.

In addition to altered connectivity with the PCC, individuals with MCI who had increased difficulty with lane maintenance exhibited reduced connectivity within the cerebellum as well as reduced connectivity between the cerebellum and the middle and inferior temporal cortex. The cerebellum is a region important for motor control [40] as well as sustained attention [39], both of which are important functions involved in car following [10,35]. Furthermore, previous car following studies [10,35] have supported the importance of the cerebellum in successful performance on this task. Thus, altered connectivity within the cerebellum among patients with MCI may reflect reduced motor control and sustained attention, ultimately leading to greater steering difficulty (SD in steering) and increased lane deviations. 

Although the current results provide important findings on functional brain changes in MCI during a complex, real-world task, there are a few methodological limitations. First, driving simulation has been noted to be being less realistic than on-road assessments and real-world driving [41,42]. This is particularly relevant in the fMRI-portion of the current study, which required participants to lie supine while driving. Importantly, however, previous research has supported the use of driving simulation when evaluating lane control [43] and has shown that simulators are highly related to on-road driving performance [44]. Given that separate cohorts were used for the behavioral and fMRI portions of the study, it was not possible to determine the association between in-scanner and out-of-scanner performance. Importantly, the same car following task and similar equipment was used for both portions of the study. Nevertheless, it will be important for future work to validate in-scanner performance by confirming that it is associated with out-of-scanner performance. Another limitation of the current study involves the patient sample. MCI is a heterogeneous condition, with different subtypes. Given the small sample size in the current study, our analysis was restricted to analyzing the MCI group as a whole—including both single domain MCI and multiple domain MCI. Thus, participants had different domains of cognitive impairment. Furthermore, the etiology of MCI may be different across participants as well as the risk of progressing to AD and related dementia. It will be important for future large-scale research studies to explore the driving profile and corresponding functional brain changes associated with various subtypes of MCI, including both single and multiple-domain amnestic and non-amnestic MCI. Furthermore, future research should include a mild probable AD subgroup as well as a longitudinal follow-up component to investigate how functional connectivity changes with disease progression. Finally, the car-following task utilized was unidimensional in space (simulated driving along a straight road) and did not involve situations that provided a range of complexity levels. Given the subtle nature of the cognitive and functional impairment associated with MCI, important additional information may be revealed by investigating both driving performance and associated functional brain changes over a range of routine to cognitively demanding aspects of driving.

## 5. Conclusions

Consistent with previous research, individuals with MCI demonstrated minor difficulty with lane maintenance, exhibiting increased steering variability compared to cognitively healthy controls during a car following task. In addition, a potential neural substrate of behavioral driving impairment in MCI was identified. Individuals with MCI exhibited reduced connectivity within the fronto-parietal network, which is important for visuospatial attention and driving [9,32]; as well as between regions involved in cognitive control (i.e., medial frontal cortex) and regions important for visual processing, including occipital and parietal regions. Thus, patients with MCI showed reduced connectivity, and information sharing, between regions involved in visual attention, visual processing, cognitive control, and performance monitoring. Individuals with MCI who experienced greater difficulty in lane maintenance (i.e., increased steering variability and lane deviations) showed increased connectivity between the PCC, a region of the DMN, and regions within the inferior frontal gyrus. This indicates that some individuals with MCI may exhibit failed or decreased attenuation of components of the DMN, leading to interference with task-relevant networks and increased driving errors. The present work is the first to use fMRI to characterize the relationship between cognitive impairment, brain functional connectivity, and performance on a cognitively complex task–driving. This provides further support that individuals with MCI may exhibit modest difficulty performing complex daily activities, including driving, and highlights the importance of further research investigating the use of functional connectivity as a biomarker of MCI and AD.

## Figures and Tables

**Table 1:** Demographic information of patients with MCI (*n* = 24) and healthy older control participants (*n* = 20) who completed the behavioral (out-of-scanner) session.

	Healthy Controls (*n* = 20)	MCI Patients (*n* = 24)	*p*-Value
Age, years	66.7 ± 8.2	66.5 ± 9.5	0.924
Education, years	16.7 ± 2.0	15.0 ± 2.6	0.072
Sex, *n* (%) male	14 (70.0%)	14 (58.3%)	0.350
Driving expereince, years	44.3 ± 12.6	45.8 ± 10.4	0.711
Driving experience, hours/week	6.8 ± 6.2	6.3 ± 4.6	0.945
Self-reported accidents	1.8 ± 2.4	1.6 ± 1.4	0.946
MoCA total score (/30)	27.9 ± 1.2	23.8 ± 1.9	<0.001

MCI, mild cognitive impairment; MoCA, Montreal Cognitive Assessment; *n*, number of participants.

**Table 2:** Car following simulation outcomes for patients with MCI (*n* = 24) and healthy older control participants (*n* = 20) for the behavioral session.

	Healthy Controls (*n* = 20)	MCI Patients (*n* = 24)	*p*-Value
Centerline crossings	0.3 (0.6)	0.9 (2.7)	0.608
Roadedge excursions	0.4 (0.9)	2.3 (4.7)	0.084
Lane deviations	0.7 (1.2)	3.2 (6.5)	0.171
Collisions	0 (0)	0.04 (0.2)	0.361
Speed exceedances	0.6 (1.0)	0.9 (1.1)	0.154
Total errors	1.3 (1.4)	4.2 (7.3)	0.147
SDLP, m	0.3 (0.1)	0.4 (0.2)	0.409
SD in steering, degrees	2.8 (0.3)	3.2 (0.8)	0.018
SD in speed, km/h	9.0 (2.2)	10.5 (4.5)	0.157
SD in range from car, m	23.9 (11.3)	21.2 (16.2)	0.126

MCI, mild cognitive impairment; SD, standard deviation; SDLP, standard deviation in lane position. Lane deviations: sum of centerline crossings and road edge excursions. Total errors: sum of lane deviations, collisions, and speed exceedances.

**Table 3:** Demographic information of patients with MCI who completed the behavioral session (*n* = 24) and the fMRI session (*n* = 15).

	Behavioral MCI Patients (*n* = 24)	fMRI MCI Patients (*n* = 15)	fMRI Healthy Controls
Age, years	66.5 ± 9.5	67.0 ± 9.3	65.1 ± 9.0
Education, years	15.0 ± 2.6	16.1 ± 4.2	17.0 ± 2.5
Sex, *n* (%) male	14 (58.3%)	12 (80%)	11 (73%)
Driving expereince, years	45.8 ± 10.4	47.5 ± 9.0	47.3 ± 9.5
Driving experience, hours/week	6.3 ± 4.6	8.1 ± 4.7	6.8 ± 4.7
Self-reported accidents	1.6 ± 1.4	1.5 ± 1.4	1.3 ± 1.3
MoCA total score (/30)	23.8 ± 1.9	24.7 ± 1.7	28.2 ± 1.0

MCI, mild cognitive impairment; MoCA, Montreal Cognitive Assessment; *n*, number of participants.

## Appendix A

**Table A1:** Areas of reduced functional connectivity observed between individuals with MCI compared to cognitively healthy controls.

Region 1	MNI Coordinates			Region 2	MNI Coordinates			Bootstrap Ratio
	X	Y	Z		X	Y	Z	
L Inf Parietal	−42	−50	48	L Mid Frontal	−36	30	36	−3.77
R Sup Parietal	28	−62	62	R Mid Frontal	36	30	36	−4.08
R Inf Parietal	46	−48	54	R Mid Frontal	36	30	36	−3.74
R Precuneus	8	−60	44	R Mid Frontal	36	30	36	−4.06
R Cuneus	14	−82	30	R Frontal Inf Tri	50	28	18	−3.88
R Sup Occipital	24	−84	30	R Frontal Inf Tri	50	28	18	−3.70
R Supramarginal	58	−34	36	R Frontal Inf Tri	50	28	18	−4.19
Vermis	2	−68	−14	R Frontal Inf Tri	50	28	18	−3.96
Vermis	2	−72	−24	R Frontal Inf Tri	50	28	18	−3.66
R Sup Occipital	24	−84	30	L Sup Med Frontal	−4	46	30	−4.10
R Angular	46	−62	40	L Sup Med Frontal	−4	46	30	−4.35
R Angular	46	−62	40	R Sup Med Frontal	8	50	30	−4.06
R Amygdala	28	−4	−14	L Amygdala	−22	−4	−14	−3.66
L Caudate	−10	10	8	R Cuneus	14	−76	12	−4.02
L Caudate	−10	10	8	L Cuneus	−8	−82	30	−4.28
R Caudate	14	10	8	L Cuneus	−8	−82	30	−3.88
R Frontal Inf Oper	50	12	22	Vermis	2	−68	−14	−4.10
L Cerebellum	−30	−62	−46	Vermis	2	−56	−34	−4.17
L Cerebellum	−26	−56	−46	Vermis	2	−56	−34	−3.72
L Cerebellum	−10	−50	−46	Vermis	2	−56	−34	−5.02

Inf, inferior; L, left; Mid, middle; R, right; Sup, superior.

**Table A2:** Areas of altered functional connectivity observed among individuals with MCI associated with increased lane deviations.

Region 1	MNI Coordinates			Region 2	MNI Coordinates			Bootstrap Ratio
	X	Y	Z		X	Y	Z	
R Post Cingulum	8	−44	22	R Frontal Inf Oper	50	12	22	4.09
R Post Cingulum	8	−44	22	R Frontal Inf Tri	50	28	18	4.49
R Lingual	14	−68	−2	R Sup Occipital	24	−84	30	3.81
Vermis	2	−54	−6	R Med Orb Frontal	8	50	−6	3.80
R Mid Temporal	58	−38	−2	R Post Cingulum	8	−44	22	4.26
R Cerebellum	28	−34	−42	L Heschl	−38	−22	12	3.99
R Cerebellum	12	−54	−46	Vermis	2	−68	−34	−4.34

Inf, inferior; L, left; Med, medial; Mid, middle; Post, posterior; R, right; Sup, superior.

**Table A3:** Areas of altered functional connectivity observed among individuals with MCI associated with standard deviation in steering.

Region 1	MNI Coordinates			Region 2	MNI Coordinates			Bootstrap Ratio
	X	Y	Z		X	Y	Z	
Vermis	2	−40	−20	L Sup Med Frontal	−4	46	30	4.62
Vermis	2	−40	−20	R Sup Med Frontal	8	50	30	4.27
R Inf Oper Frontal	50	12	22	R Post Cingulum	8	−44	22	4.06
R Inf Tri Frontal	50	28	18	R Post Cingulum	8	−44	22	4.82
R Mid Temporal	58	−38	−2	R Post Cingulum	8	−44	22	3.92
L Rolandic Oper	−48	−10	18	L Angular	−44	−62	36	−3.47
R Sup Orb Frontal	18	44	−14	L Caudate	−10	10	8	3.95
R Inf Orb Frontal	40	30	−10	R Sup Temporal Pole	50	12	−14	−4.05
L Cerebellum	−22	−62	−20	L Mid Temporal	−58	−38	−2	−4.08
R Cerebellum	18	−48	−20	R Mid Temporal	58	−38	−2	−3.37
L Cerebellum	−14	−48	−14	L Inf Temporal	−52	−32	−24	−3.37
R Cerebellum	28	−60	−24	L Cerebellum Crus	−26	−76	−38	−4.77
R Cerebellum	28	−60	−46	L Cerebellum Crus	−36	−68	−28	−3.80
R Cerebellum Crus	36	−68	−28	R Cerebellum Crus	34	−72	−38	−4.46
L Cerebellum	−10	−50	−46	R Cerebellum Crus	34	−72	−38	−4.54
L Cerebellum	−22	−62	−20	L Cerebellum Crus	−26	−76	−38	−3.55
Vermis	2	−68	−34	L Cerebellum Crus	−26	−76	−38	−4.06
Vermis	2	−54	−6	R Cerebellum Crus	34	−72	−38	−3.35
L Cerebellum	−14	−48	−14	R Cerebellum Crus	34	−72	−38	−4.46
L Cerebellum	−26	−56	−46	R Cerebellum Crus	34	−72	−38	−4.30
Vermis	2	−68	−34	R Cerebellum Crus	34	−72	−38	−5.43
R Cerebellum	34	−62	−46	L Cerebellum	−14	−48	−14	−3.92
Vermis	2	−40	−20	R Cerebellum	34	−62	−46	−3.63
Vermis	2	−54	−6	R Cerebellum	34	−62	−46	−3.34
Vermis	2	−68	−34	R Cerebellum	34	−62	−46	−3.39
L Rolandic Oper	−48	−10	18	L Cerebellum	−22	−34	−42	4.39
R Sup Frontal	20	28	44	Vermis	2	−40	−20	3.49
R Mid Frontal	36	30	36	Vermis	2	−40	−20	3.70

Inf, inferior; L, left; Med, medial; Mid, middle; Post, posterior; R, right; Sup, superior.
